# A green route for the cross-coupling of azide anions with aryl halides under both base and ligand-free conditions: exceptional performance of a Cu_2_O–CuO–Cu–C nanocomposite

**DOI:** 10.1039/c8ra04608e

**Published:** 2018-07-18

**Authors:** Morteza Karimzadeh, Khodabakhsh Niknam, Neda Manouchehri, Dariush Tarokh

**Affiliations:** Department of Chemistry, Faculty of Sciences, Persian Gulf University Bushehr 75169 Iran niknam@pgu.ac.ir khniknam@gmail.com

## Abstract

A convenient, inexpensive and effective route for the preparation of a Cu_2_O–CuO–Cu–C nanocomposite is described here by applying Cu(ii) as a source of copper. Characterization of the nanocomposite was performed with X-ray diffraction (XRD), Fourier-transform infrared spectroscopy (FT-IR), transmission electron microscopy (TEM), high-resolution TEM (HR-TEM), field emission scanning electron microscopy (FE-SEM), X-ray photoelectron spectroscopy (XPS), and energy-dispersive X-ray spectroscopy (EDX). Analysis of the data showed that the particles of the nanocomposite are uniformly distributed and show high catalytic activity in the cross-coupling of sodium azide with various aryl iodides and bromides. This nanocomposite has a high level of performance, and even led to the synthesis of the products at room temperature. In addition, this is the first report of the synthesis of aryl azides under both base- and ligand-free conditions. For the first time, both ligand- and base-free conditions were applied for the synthesis of aryl azides, which implies exceptional performance of the Cu_2_O–CuO–Cu–C nanocomposite. Simultaneous removal of the base and ligand in a green solvent is the main advantage of this reaction. Unfortunately, aryl bromides and aryl iodides with electron-withdrawing functional groups in their scaffold did not give the desired aryl azides.

## Abstract 2

Ligand-free and base-free conditions were used for the preparation of aryl azides using the cross-coupling of sodium azide and aryl halides catalysed by Cu_2_O–CuO–Cu–C.

## Graphical abstract

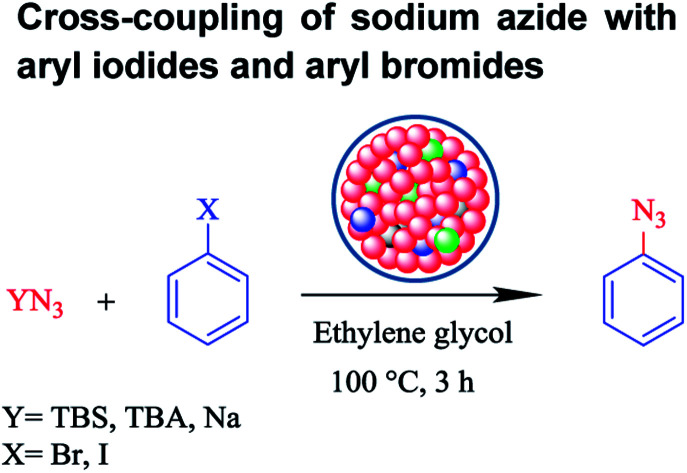

## Introduction

Aryl azides are extensively used in bioactive molecules as synthetic motifs due to their wide range of usages.^1–3^ These compounds contain special functional groups with the capability to get involved in the formation of nitrenes and insert different heteroatoms. In addition, their affectivity as optical sensors has already been described.^4^ These compounds have beneficial effects in photography,^5^ dendrimers with conducting capability^6^ and light-rendered energizing polymers.^7^ Thus, obtaining an effective route to produce such compounds is highly desirable.

Thus far, there aren't many synthetic routes for synthesizing aryl azides. The older method is deazotisation of aromatic amines.^8^ Using this method, obtaining the desired product is really difficult when more than one amino group is present on the aromatic scaffold. An alternative path for producing aryl azides is nucleophilic aromatic substitution reaction of NaN_3_ with activated aryl fluorides and chlorides.^9^ In general, such methods are not appropriate if electron-donating substituents are present on the aromatic scaffolds. Aryl azides can also be produced from the respective aryl boronic acids.^10^ Nonetheless, this procedure is annulled due to the low availability of aryl boronic acid derivatives. Accordingly, reactions of sodium azide with aryl halides are favorable. It is notable that a confusing variety of reports have been obtained in relation to achieving aryl azides or amines in the cross-coupling of sodium azide with aryl halides.

Ma and co-workers recently published an article in which a CuI–proline mixture catalyzed the synthesis of aryl and alkenyl azides in a green medium.^11^ Liang and co-workers showed a rapid procedure for the synthesis of aryl azides by applying CuI/DMEDA as a catalytic system.^12,13^ Tatcher reported solvent switching from 7EtOH/3H_2_O to DMSO/2EtOH along with the use of excess quantities of sodium azide, stoichiometric quantities of base and CuI/l-proline, which led to the formation of aryl amines instead of the corresponding aryl azides. Aryl amines were produced due to the instability of the aryl azides at higher temperatures.^14,15^ Aryl amines have recently been synthesized by the substitution of halides with higher quantities of sodium azide and by applying Cu_2_O/DMEDA as a catalytic system.^16^ Bewildering arrays of reports and the formation of different products led to the selective synthesis of aryl azides using the obtained nanocomposites.^17,18^

**Fig. 1:**
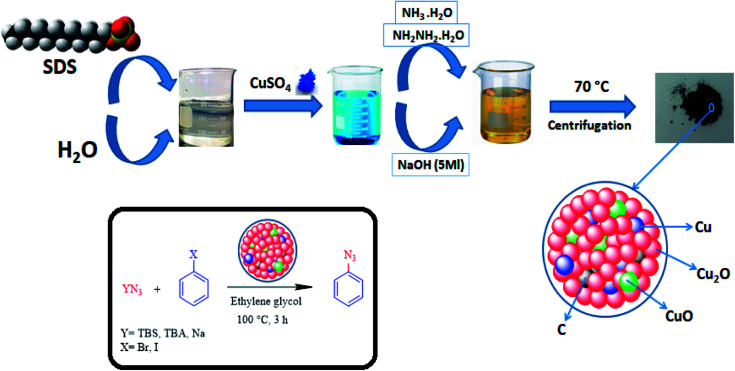
Schematic representation of the preparation of the Cu_2_O–CuO–Cu–C nanocomposite and its application in the cross-coupling of azide anions with aryl halides.

Metal-based nanocomposite materials have been shown tremendous interest in the chemical industry due to them having fascinating potential physical and chemical properties.^19^ These modulated materials have special structural scaffolds, and their characteristics are mostly dependent on the morphology, size, composition, and architecture of metal-based nano-composites, indicating that they are more advantageous in comparison with simple copper oxides.^20,21^ Therefore, notable efforts have been introduced to form different metal-based nanocomposites for particular applications.^22–29^ In an attempt to improve on the Helquist conditions, a Cu_2_O–CuO–Cu–C nanocomposite will be introduced in the following to introduce the first selective synthesis of aryl azides under both base-free and ligand-free conditions (Fig. 1).

## Results and discussion

**Fig. 2:**
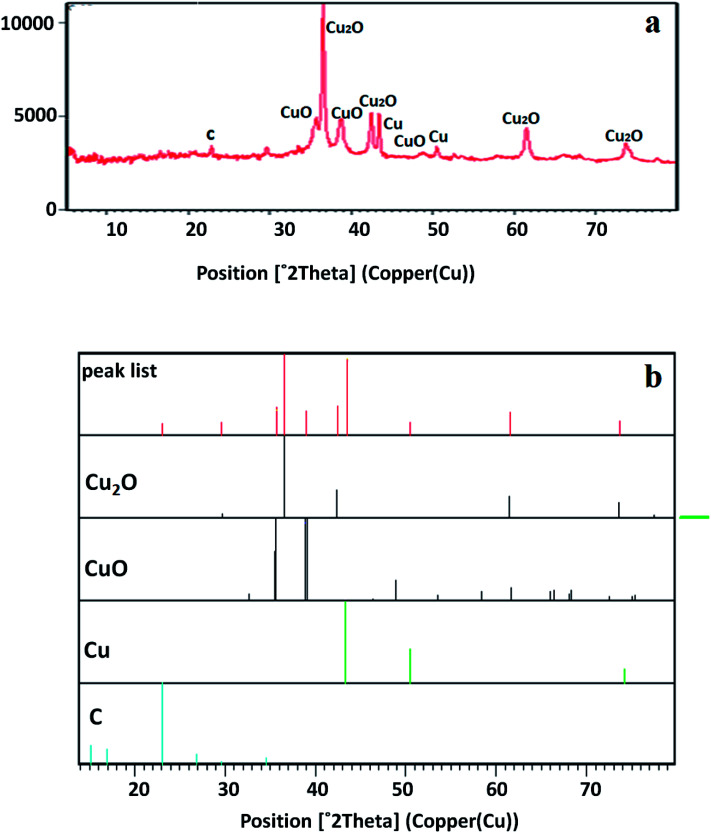
XRD pattern of the Cu_2_O–CuO–Cu–C nanocomposite compared with Cu_2_O, CuO, Cu, and C.

The crystal structure of the copper-based nanocomposite was studied by XRD analysis (Fig. 2). The characteristic peaks observed at 2*θ* = 29.63° (110), 36.52° (111), 42.40° (200), 61.48° (220), and 73.64° (311) are ascribed to the cuprite phase of Cu_2_O. Two other small peaks presented at 2*θ* = 35.57° and 38.69° are attributed to the melaconite phase of CuO. The other two peaks observed at 2*θ* = 43.37° and 50.48° are indicative of the presence of metallic Cu. The only small peak that appeared at 2*θ* = 22.84° is representative of C with carbolite phase. In addition, the strong and sharp peaks showed the crystalline nature of the copper-based nanocomposite. As it is demonstrated, the height of the peaks in the case of Cu_2_O is much higher than that of the CuO, metallic Cu, and C peaks, indicating that the nanocomposite is mainly composed of Cu_2_O. Furthermore, the amount of carbolite phase in the nanocomposite is negligible when the height of this peak is compared to the others, indicating that a very small amount of C is present in the nanocomposite.^30,31^

**Fig. 3:**
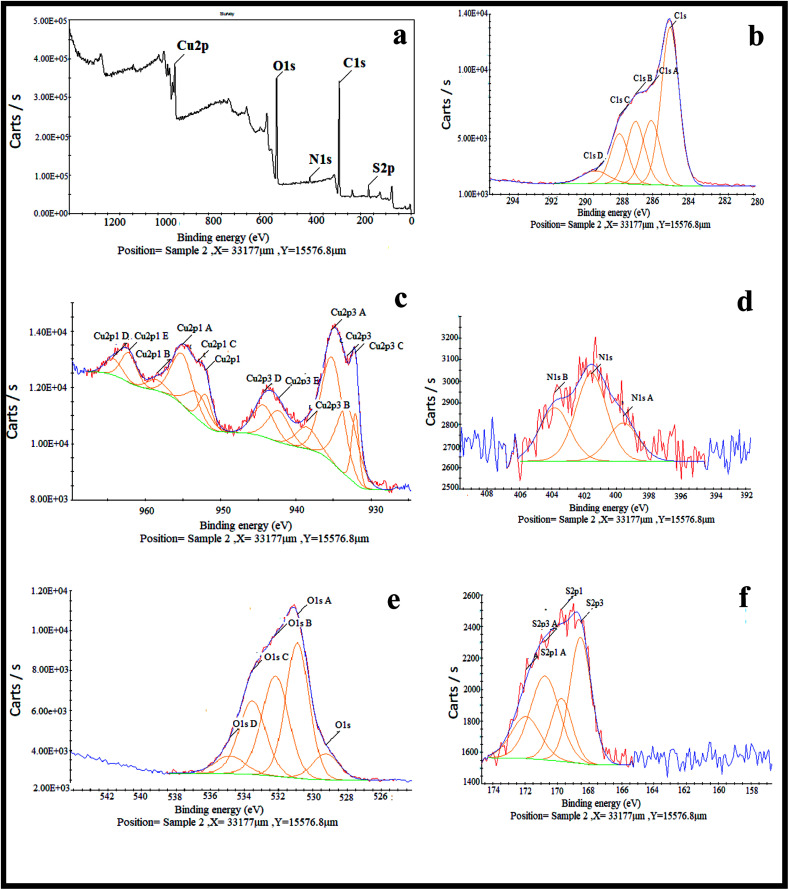
XPS spectra of (a) the Cu_2_O–CuO–Cu–C nanocomposite, (b) the C1s scan, (c) the Cu2p scan, (d) the N1s scan, (e) the O1s scan and (f) the S2p scan.

XPS was used to study the chemical composition and oxidation state of the nanocomposite. Looking at Fig. 3a–f indicates that the nanocomposite contains S, C, N, O and Cu elements. Based on previous reports, the peaks that appear at 932.09 and 933.17 eV labelled as Cu^2p3^ and Cu^2p3c^ were ascribed to Cu_2_O/Cu (Cu^+^/Cu^0^), because differentiation between the binding energies of Cu_2_O and Cu by XPS is difficult. Another peak that appears at 935.21 eV labelled as Cu^2p3A^ was assigned to CuO, even though this energy is slightly higher than the reported values. It is notable that the co-existence of Cu_2_O, metallic Cu and CuO has already been identified by XRD analysis. The peak observed at 938.55 eV labelled as Cu^2p3B^ is associated with Cu(OH)_2_. Five different peaks at 529.73, 531.39, 532.69, 534.04, and 535.35 eV are the reason for the broadened peak of O1s, in which the peak appears at 529.73 related with the O1s of Cu_2_O. The O1s of CuO appeared at 534.04 eV, and the other peaks may result from the O1s of other components containing an oxygen atom in their moiety, like H_2_O, OH, carbonate or sulfate. There are five different binding energies in the range of 284.46–288.91 eV observed for the C1s core level, in which the main component associated with 284.46 eV is consistent with C–C species. There are other peaks represented in the XPS spectrum corresponding to small amounts of nitrogen and sulfur atoms having values of 401.45 and 169.38 eV, respectively, for broadening of the N1s and S2p peaks in the composition of the nanocomposite.^30,31^

**Fig. 4:**
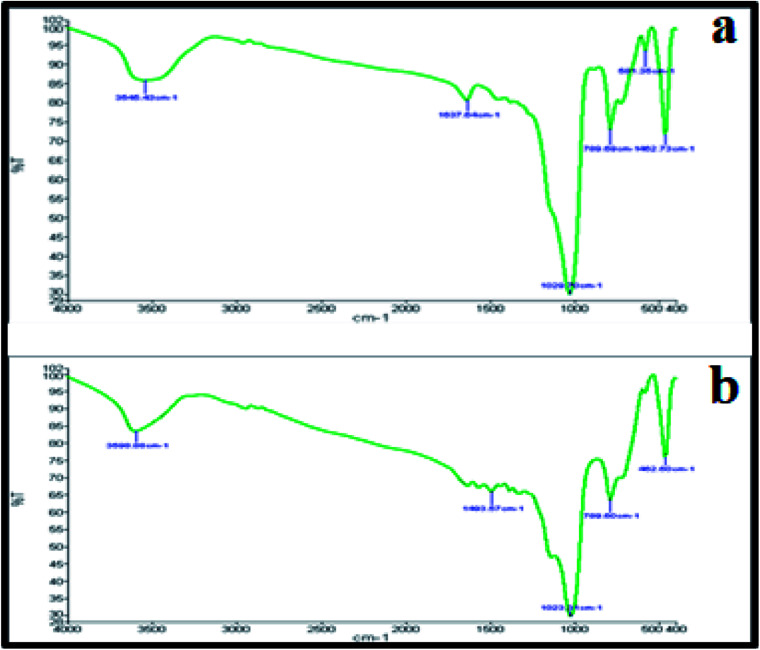
FT-IR spectra of the Cu_2_O–CuO–Cu–C nanocomposite: (a) before the reaction and (b) after the reaction.

Fig. 4a and b represent the FT-IR spectrum of the studied nanocomposite before and after the reaction. The important absorption bands are observed at 461.87, 580.27, 790.35, 1020.22, 1634.84 and 3553.44 cm^−1^. The three absorption bands that appeared at 461.87, 580.27 and 790.35 cm^−1^ are correlated with Cu–O stretching vibration. The broad and strong band that appeared at 1020.22 cm^−1^ may be associated with C–C stretching vibration and O–H bending vibration. The peak that appeared at 1634.84 is an indicator of water molecules present in the nanocomposite. In addition, the O–H stretching vibration shows a broad band at around 3553.44 cm^−1^. Comparing the FT-IR spectra of the catalyst before and after the reaction showed that there is not that much difference between them, indicating the stability of the catalyst.

**Fig. 5:**
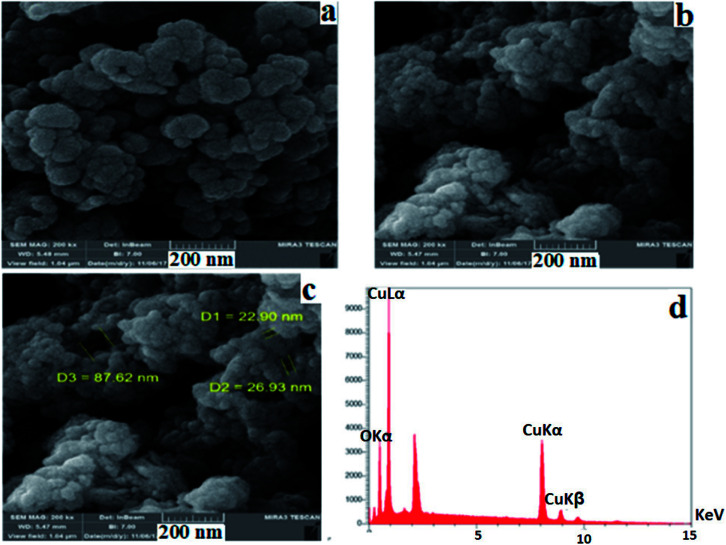
(a–c) SEM images and (d) EDX spectrum of the Cu_2_O–CuO–Cu–C nanocomposite.

Fig. 5a–c show the FE-SEM pictures of the obtained nanocomposite with a rough surface, in which uniform hollow-like spheres were distributed in different sizes with the diameter of the hollow structures ranging from 22.90 to 87.62 nm. These hollow structures are composed of small nanoparticles, making holes on the surface. EDAX analysis (Fig. 5d) shows that the copper based nanocomposite contains Cu and O elements, and other elements (C, S, and N elements) could not be detected by EDAX, because the amounts of these elements in the nanocomposite structure are very low, with the total weight percent of copper and oxygen atoms being, respectively, 69.45% and 30.55%.

**Fig. 6:**
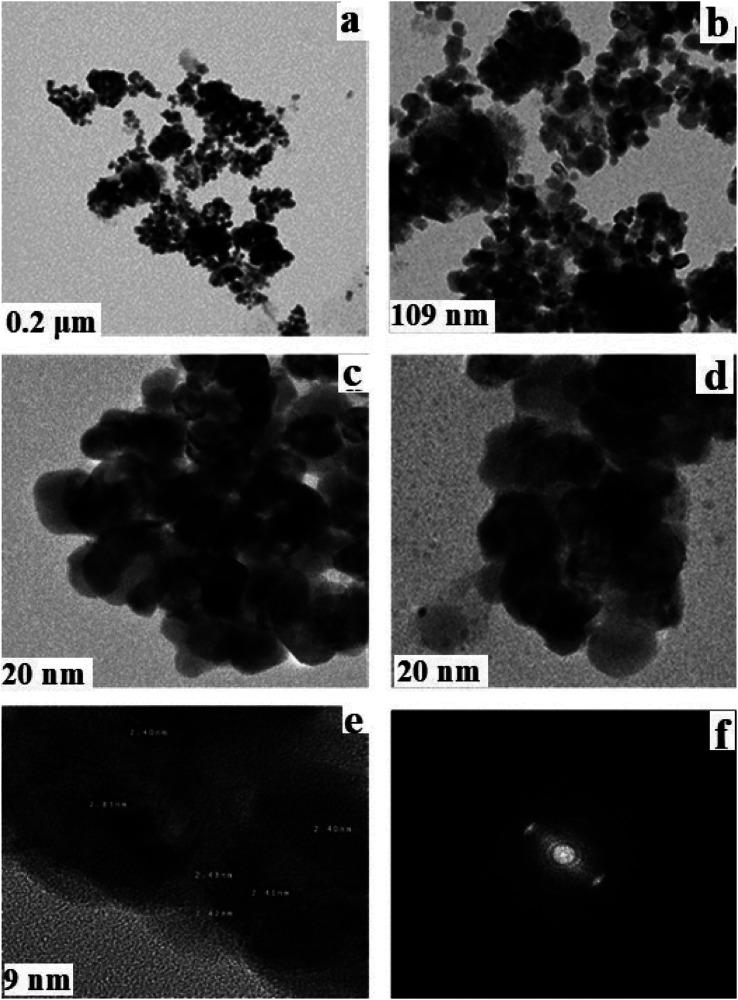
(a–d) TEM, (e) HR-TEM and (f) SAED images of the Cu_2_O–CuO–Cu–C nanocomposite.

Fig. 6a–f depict the corresponding TEM, high-resolution TEM and selected area electron diffraction pictures (SAED) of the copper-based nanocomposite. The morphology of the nanocomposite showed hardly any hollow spherical structures, though some could be distinguished with careful observation. Approximately, the particles in the resulting nanocomposite are almost the same size and uniformly distributed. The fringe spaces of 2.40, 2.41, and 2.43 nm indicated in the HR-TEM image relate well with that of polycrystalline Cu_2_O–CuO–Cu. The SAED pattern completely agrees with the XRD pattern showing the expected (111) and (200) diffraction peaks in accordance with the crystal planes of Cu_2_O, because the main component of the nanocomposite is Cu_2_O. Therefore, the diffraction rings observed in the SAED pattern match well with the corresponding crystal planes of the nanocomposite, indicating that the resultant nanocomposite is not completely pure, and the Cu_2_O contains some metallic copper and cupric oxide impurities.

Herein, the coupling of sodium azide and aryl halides has been investigated by applying a catalytic mixture of the copper-based nanocomposite and amino acids under different conditions. Optimization was started by applying iodobenzene (1 eq.) and sodium azide (1 eq.) as model substrates (Table 1). These compounds were mixed with 0.1 eq. of NaOH as a base, 0.1 eq. of l-proline as a ligand, and 0.02 g of the copper nanocomposite in EtOH as a solvent at 100 °C. Under these conditions, the corresponding azide was selectively obtained in 95% yield after 100 min and the formation of aryl amines failed. Then, the reaction was repeated without the base. Interestingly, the yield of product did not change in the same time, indicating that the base does not have any role in this reaction. Afterwards, the same reaction was repeated without the base and ligand. Strangely enough, significant results were obtained in this regard, and the yield of azidobenzene did not differ from the previous one, indicating the exceptional role of the catalyst in the difficult coupling of sodium azide with iodobenzene. Optimization of the reaction continued by changing the solvent from EtOH to water, ethylene glycol and PEG-400. The reaction did not work when water was used as the reaction medium, but in the case of PEG-400 (60%) a smaller amount of product was achieved under the same conditions. By applying ethylene glycol instead of EtOH, there was no difference in conversion to azidobenzene, and for the following two reasons, ethylene glycol was chosen as the best solvent: easier separation of the products and the required reaction temperature. The same reaction was investigated at room temperature, and surprisingly 50% conversion was obtained after just 3 h, but a longer reaction time (3 days) was needed for the reaction to proceed to complete conversion. It should be noted that lowering the quantity of catalyst from 0.02 g to 0.01 g led to a lower amount of product. Therefore, 0.02 g of the Cu-nanocomposite was selected as the optimized amount. It is notable that this catalyst is classified as the first one that catalyses this cross-coupling reaction without the use of a base, and ligand at room temperature. To the best of our knowledge, there is no reported article in which one of these conditions becomes accessible.

**Table 1:** Optimization of the cross-coupling reaction between NaN_3_ and iodobenzene catalyzed by Cu_2_O–CuO–Cu–C

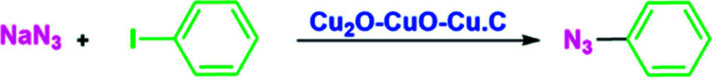							
Entry	Solvent^a^	Time^a^ (h)	Temperature^a^ (°C)	NaOH^a^	l-Proline^a^ (mol%)	Catalyst^a^ (mol%)	Conversion^b^ (%)
1	EtOH	100 min	100	10 mol%	10 mL%	0.02 g	95%
2	EtOH	100 min	100	—	10 mL%	0.02 g	95%
3	EtOH	100 min	100	—	—	0.02 g	95%
4	H_2_O	100 min	100	—	—	0.02 g	—
5	EG	100 min	100	—	—	0.02 g	95%
6	PEG-400	100 min	100	—	—	0.02 g	60%
7	EG	180 min	r.t.	—	—	0.02 g	50%
8	EG	3 days	r.t.	—	—	0.02 g	99%
9	EG	100 min	100	—	—	0.01 g	65%
**10**	**EG**	**180 min**	**100**	—	—	**0.02 g**	**99%**

a Reaction conditions: iodobenzene (1 mmol), NaN_3_ (2 mmol), catalyst.

b Conversion.

To explore the generality and scope of the reaction, various aryl halides (iodides, bromides and chlorides) were reacted with different sources of azides (sodium azide, trimethylsilyl azide, tetrabutylammonium azide). For this process, the reaction of bromobenzene and 4-bromoanisole with sodium azide under the optimized conditions was checked and the desired amounts of the products were achieved at 100 °C (Table 2). The same reactions were investigated at room temperature, but unfortunately aryl bromides were inactive under reduced temperatures, unlike iodobenzene. In addition, one of the main drawbacks of this reaction was the lack of formation of aryl azides possessing strong electron withdrawing groups. Another drawback is caused by the aryl chlorides not being able to react with different sources of azides under the optimized conditions. A closer look at Table 2 indicates that the aryl iodides and aryl bromides containing electron-donating groups in the *meta* and *para* position gave good to excellent results. Two other nucleophilic azide sources alternative to sodium azide were also used in aryl azide preparation (Table 2, entries 12 and 13). Unfortunately, these materials were almost inactive when reacted with iodobenzene, and smaller amounts of products were obtained under the optimized conditions.

**Table 2:** Generality of the cross-coupling of aryl halides with an azide source catalyzed using Cu_2_O–CuO–Cu–C

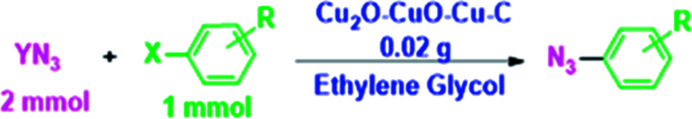					
Entry	Aryl halide R	Aryl halide X	Azide source Y	Product R	Yield^a^ (%)
1	H	I	NaN_3_	H	99%
2	4-I	I	NaN_3_	4-N_3_	85%
3	4-Br	Br	NaN_3_	4-N_3_	76%
4	4-OMe	Br	NaN_3_	4-OMe	85%
5	4-OMe	I	NaN_3_	4-OMe	99%
6	H	Cl	NaN_3_	H	—
7	4-SO_2_Me	Br	NaN_3_	4-SO_2_Me	—
8	3-OMe	I	NaN_3_	3-OMe	90%
9	3-OMe	Br	NaN_3_	3-OMe	78%
10	4-SMe	Br	NaN_3_	4-SMe	82%
11	4-CN	Br	NaN_3_	4-CN	—
12	H	I	TMSN_3_	H	8%^b^
13	H	I	TBAN_3_	H	14%^b^

a Reaction conditions: azide source (2 mmol), aryl halide (1 mmol), catalyst (0.02 g), ethylene glycol (3 mL), 100 °C, 3 h.

b Data reported based on the conversion.

There are different reported mechanisms for such Ullmann-type cross-coupling. Herein, the detailed mechanism for the cross-coupling of the azide anion with aryl halides is still not completely understood, although an obvious parallelism can be taken into account with other reported articles. Therefore, we propose oxidative addition of aryl halides onto the surface of the Cu_2_O–CuO–Cu–C nanocomposite, in which Cu_2_O acts as the main active species. In the next step, replacement of the azide anion with halides can be done. Therefore, NaX is released, and then the liberation of the aryl azides takes place using a reductive elimination pathway to recover the catalyst. In addition, the presence of carbon in the catalyst can be helpful in reducing the aggregation of the active copper species. It is also probable that the reaction is promoted by π-complex formation of the aryl halides with the active copper species.^32,33^

## Conclusions

An exceptional and inexpensive Cu_2_O–CuO–Cu–C nanocomposite was introduced as a catalyst for the synthesis of aryl azides from the corresponding aryl halides and sodium azide. The catalyst had comparable activity in comparison with its counterparts in sodium azide and aryl halide cross-coupling. The applicability of the catalyst without the need for a ligand or base is the most important feature of this difficult cross-coupling. Different aryl iodides and bromides were tested in this reaction and good to excellent results for the production of the respective aryl azides were obtained. Aryl chlorides and other aryl halides with electron-withdrawing groups in their moieties were not reactive under these conditions.

## Experimental section

All of the starting materials were purchased from Sigma-Aldrich and used without further purification. X-ray diffraction patterns were obtained by a Panalytical X'pert PRO X-ray Diffractometer using a CuK target from the Netherlands. XPS analysis was achieved by a Thermofisher Scientific K-Alpha instrument. FT-IR measurements were performed on a Spectrum Two FT-IR spectrometer from PerkinElmer. The morphology and particle size of CuO–Cu_2_O–Cu–C were studied by field emission scanning electron microscopy using a MIRA3 instrument from TESCAN. Energy dispersive X-ray spectroscopy was performed with the same instrument. The diameter of the particles of CuO–Cu_2_O–Cu–C was assessed with the high-resolution transmission electron microscopy technique with a Zeiss-EM10C-100 kV instrument from Germany.

### Procedure for the synthesis of the Cu_2_O–CuO–Cu–C catalyst

For the synthesis of the desired Cu-based nanocomposite, 0.36 g of sodium dodecyl sulfate (SDS) was dissolved in 90 mL of deionized water and stirred at room temperature for 30 min. Then, 2 mL of cupric sulfate with a concentration of 0.1 g mL^−1^ was added to the above solution and stirred for another 20 min. Then, 0.08 mL of ammonia (13 M) and 0.3 mL of sodium hydroxide (5 M) were sequentially added to the same mixture after each 20 min. A change in colour of the solution from clear to opaque was observed. Finally, 0.3 mL of hydrazine solution (64 wt%) was poured into the solution as a reducing agent. As soon as the hydrazine was added to the solution, a large number of bubbles were formed in the solution. After the completion of the reaction, Cu_2_O–CuO–Cu–C was easily separated by centrifugation and washed several times with ethanol, water and mixture of the two, and then filtrated and dried at 70 °C for 24 h under vacuum.

### Procedure for the synthesis of aryl azides

For the preparation of azidobenzene, 1 mmol of iodobenzene and 2 mmol of sodium azide were dissolved in ethylene glycol in a flask tube under stirring, and then 0.02 g of the Cu_2_O–CuO–Cu–C nanocomposite was added to the same mixture and heated at 100 °C for 3 h. A work-up procedure was performed using water and a mixture of hexane/ethyl acetate after evaluating complete conversion to the corresponding azide using TLC. After evaporation of the organic solvents, the desired azidobenzene was obtained. For the synthesis of the other derivatives, the same procedure was applied, though in some cases purification of the desired products was performed by applying column chromatography.

## Conflicts of interest

There are no conflicts to declare.

## Supplementary Material
